# Midkine: The Who, What, Where, and When of a Promising Neurotrophic Therapy for Perinatal Brain Injury

**DOI:** 10.3389/fneur.2020.568814

**Published:** 2020-10-22

**Authors:** Emily Ross-Munro, Faith Kwa, Jenny Kreiner, Madhavi Khore, Suzanne L. Miller, Mary Tolcos, Bobbi Fleiss, David W. Walker

**Affiliations:** ^1^Neurodevelopment in Health and Disease Research Program, School of Health and Biomedical Sciences, Royal Melbourne Institute of Technology (RMIT), Melbourne, VIC, Australia; ^2^School of Health Sciences, Swinburne University of Technology, Melbourne, VIC, Australia; ^3^The Ritchie Centre, Hudson Institute of Medical Research, Monash University, Clayton, VIC, Australia; ^4^Neurodiderot, Inserm U1141, Universita de Paris, Paris, France

**Keywords:** midkine, neurotrophins, hypoxia, neuroinflammation, perinatal brain damage, preterm birth

## Abstract

Midkine (MK) is a small secreted heparin-binding protein highly expressed during embryonic/fetal development which, through interactions with multiple cell surface receptors promotes growth through effects on cell proliferation, migration, and differentiation. MK is upregulated in the adult central nervous system (CNS) after multiple types of experimental injury and has neuroprotective and neuroregenerative properties. The potential for MK as a therapy for developmental brain injury is largely unknown. This review discusses what is known of MK's expression and actions in the developing brain, areas for future research, and the potential for using MK as a therapeutic agent to ameliorate the effects of brain damage caused by insults such as birth-related hypoxia and inflammation.

## Introduction

The structural and functional development of the brain depends on neurotrophic factors that drive the growth, differentiation, and migration of neural precursor cells. Midkine (MK) and pleiotrophin (PTN) are structurally and functionally related neurotrophic factors and are the only two members of the neurite growth-promoting factor family. MK is called midkine because it was originally identified as a cyto*kine* highly expressed in *mid*-gestation in many organs of the mouse, particularly the kidneys, heart, and brain (1, 2). PTN expression has a different pattern, increasing from birth and persisting into adulthood (3, 4). However, expression of MK in the adult is induced following many forms of injury, and in many forms of cancer (5), where it mediates hypoxic or inflammatory-driven cell response pathways (6, 7). Previous work has demonstrated the potential therapeutic efficacy of MK for repair and regeneration after ischemic brain damage (8) and in seizure (9), and drug addiction-related brain injuries (10). Specifically, MK has been shown to ameliorate cell death, modulate glial reactivity, and enhance proliferation and migration of neural precursor cells (8, 10). MK also promotes hypoxia-induced angiogenesis (11) and serves as a chemoattractant for leukocytes (12). However, the therapeutic potential of MK following injury to the developing CNS has yet to be explored.

In this review, we discuss the spatiotemporal expression of MK and some of its key receptors during neurodevelopment and the function of MK following injury-induced expression. Both highlight the potential for the use of MK as a treatment for perinatal brain damage. We also interrogated gene expression databases to bring together the developmental and cell specific expression of MK and where possible PTN and their receptors. Perinatal brain damage arises from events such as fetal hypoxia, birth asphyxia, exposure to *in utero* and postnatal inflammation/infection (e.g., chorioamnionitis, sepsis), and/or preterm birth. These global problems in perinatology all too often result in death or life-time disabilities (13, 14), and account for around 2.4% of the total Global Burden of Disease (15). These disabilities include cerebral palsy, mild cognitive deficits, learning difficulties, epilepsy, and pervasive behavioral deficits such as autism spectrum disorders (16). At the present time, the treatment options for perinatally acquired brain damage are very limited. The option for impending preterm birth is intrapartum use of magnesium sulfate preterm birth, but there are no postnatal therapies. The option for term-born infants diagnosed with neonatal encephalopathy (NE) linked to hypoxia-ischemia (HI), (hypoxic-ischemic encephalopathy, HIE) is hypothermia (head alone, or whole-body cooling), which is only effective if commenced within 6 h of birth and requires specialized medical facilities (17, 18). In both cases, lives are saved and outcomes are improved, but the number needed to treat (NNT) for intrapartum use of magnesium sulfate is between 42 and 74 to see a significant reduction in rates of cerebral palsy (18, 19), and the NNT is 7 for hypothermia to see a reduction in mortality and severe morbidity (20), meaning there are still many more infants that need help. This is despite considerable efforts to find adjunct therapies for use with hypothermia (21), and additional therapies for all infants. As such, there remains a strong unmet need for treatments that can be delivered easily and quickly, and over a wider window of time after birth. The focus of this review is to evaluate the potential for MK to meet this need. We surveyed publications listed on PubMed using the search term MK, and then MK coupled with cancer, brain, neuroprotection, infant, neonate, birth, HIE, prematurity, and other terms as shown in Table 1. Of the 956 papers captured, only 22 (2.3%) were linked to studies in infants, neonates, brain, and/or birth, 1 was linked to prematurity and preterm birth, and *none* was linked to HIE or other commonly associated conditions of fetal injury such as intrauterine growth restriction. This demonstrates the great paucity of studies of MK in relation to the cause and treatment of perinatally acquired brain injury in the human neonate, despite the clear potential that MK has in this regard that we will outline in this review.

**Table 1:** Details of the spectrum of publications related to Midkine (MK), highlighting the vast number of works across areas such as cancer, but the striking lack of research in the area of perinatal brain injury (PubMed search, August 20, 2020).

**Search term**	**Number of PubMed hits**	**Hits relevant to the intended search for MK in perinatal brain injury**
Midkine	956 (98 reviews)	–
Midkine AND Cancer	431 (41 reviews)	–
Midkine AND Brain	158 (14 reviews)	–
Midkine AND Neuroprotection	18 (5 reviews)	–
Midkine AND Nanoparticle	8	–
Midkine AND Inflammation	92 (12 reviews)	–
Midkine AND Infant AND Brain	3	1 (22)
Midkine AND Neonatal AND Brain	7	0
Midkine AND Perinatal AND Brain	7	0
Midkine AND Premature or Preterm birth	6	1 (23)
Midkine AND Hypoxic-ischemic Encephalopathy	0	0
Midkine AND Fetal Growth restriction OR intrauterine growth restriction	0	0

## Midkine Properties

MK is a secreted, low molecular weight (13–18 kD) basic heparin-binding protein (24). MK has a 46% homology to pleiotrophin (PTN), and both share trophic and cytokine-signaling activities. MK consists of 121 amino acids (25) and is highly endowed with the positively charged basic amino acids-arginine, lysine, and histidine (26). The mRNA and protein of mouse and human MK are similar (27), with the amino acid sequence predicted to have an 83% homology (28). The protein structure of MK is composed of N-terminal and C-terminal halves linked by five disulfide bonds. The C-terminal portion of MK holds a strong conformation-dependent heparin binding site that is needed for full expression of the neurite extension and plasminogen activator activities, but not for promoting cell survival (25, 29). It is noteworthy that the effects of MK on neuronal outgrowth and survival are highly dependent on the sulfate groups (30).

MK binds to highly sulfated structures in the glycosaminoglycan chain of proteoglycans, namely, chondroitin sulfate-E structures and the tri-sulfated structure in heparin sulfate disaccharide units prevalent on the cell surface and within the extracellular matrix (31). In fact, MK protein is the ligand for several receptor-type proteins implicated in various physiological roles as described in Table 2. Though this provides an array of potential therapeutic targets, these receptors also have several potential ligands, and this has created difficulty in defining the exact functions of MK.

**Table 2:** MK receptor-ligand binding and signaling functions.

**MK binding receptors**	**Biological functions**	**References**
Protein tyrosine phosphatase ζ (PTPZ)	◦ Promotes survival of embryonic neurons ◦ Expressed on LRP6 and apoE receptor 2 (components of reelin, Wnt, and Dickkopf receptors) neurons ◦ Antiapoptotic activity with combined effects of PP1 and PTX—inhibitors of G protein-linked signaling ◦ Promotes migration of embryonic neurons	(32, 33)
Ryudocan (Syndecan-4)	◦ Expressed abundantly in peripheral bundle nerves ◦ Interacts with tissue factor pathway inhibitor ligand ◦ Function in anticoagulant function and inhibits placental cytotrophoblasts	(34)
Syndecan-1	◦ Expressed in brain and spinal cord during earlier gestational period—E10 to E12 ◦ Promotes neurogenesis	(35)
N-syndecan (Syndecan-3)	◦ Interacts with MK during late developmental period—E14 to E16 ◦ Promote neurogenesis	(35)
Low-density lipoprotein receptor-related protein (LRP)	◦ Promotes nucleus translocation ◦ Internalizes MK in the cytoplasm-bound nucleolin, a nucleocytoplasmic shuttle protein ◦ Promotes cell survival	(36)
Neuroglycan C	◦ Promotes CG-4 cells (glial precursors for oligodendrocyte progenitor cells) ◦ Promotes elongation in glial cells	(37)
β-integrins—α_6_β1 integrin and α_4_β1 integrin	◦α_4_β1 integrin promotes migration of osteoblastic cells ◦α_4_β1 integrin governs haptotactic migration of osteoblastic cells ◦ Increase tyrosine phosphorylation of paxillin, a key molecule in Crk-II pathway ◦α_6_β1 integrin promotes neurite outgrowth on embryonic neurons	(38)
Lipopolysaccharide-binding (LBP) receptor—member of low-density lipoprotein receptors	◦ Activates LBP adhesion in the cytoplasm and cell surfaces ◦ Activates and acts as “shuttle protein” in translocating into nucleus ◦ Promotes tumorigenesis process	(39)
Anaplastic lymphoma kinase (ALK)	◦ Mitogenesis-potent proliferation of human endothelial cells from brain microvasculature and umbilical vein ◦ Promotes angiogenesis ◦ Activates Akt phosphorylation by 10-fold, with 2-fold increase in MAPK phosphorylation ◦ Activates NF-κB pathway ◦ Induces insulin receptor-1 to initiate mitogenesis and antiapoptosis ◦ Activates PI3K and MAPK pathways in varying ratio and response in cell types	(40–42)

*ALK, anaplastic lymphoma kinase; ApoE, apolipoprotein E; LBP, lipopolysaccharide-binding; LRP, low-density lipoprotein receptor-related protein; MAPK1, mitogen-activated protein kinase 1; MAPK3, mitogen-activated protein kinase 3; NF-kB, nuclear factor kappa-light-chain-enhancer of activated B cells; PTX, paclitaxel; PI3K, phosphatidylinositol-3-kinase; Akt, protein kinase B; PP1, protein phosphatase 1*.

## Developmental Expression of Midkine

**Figure 1:**
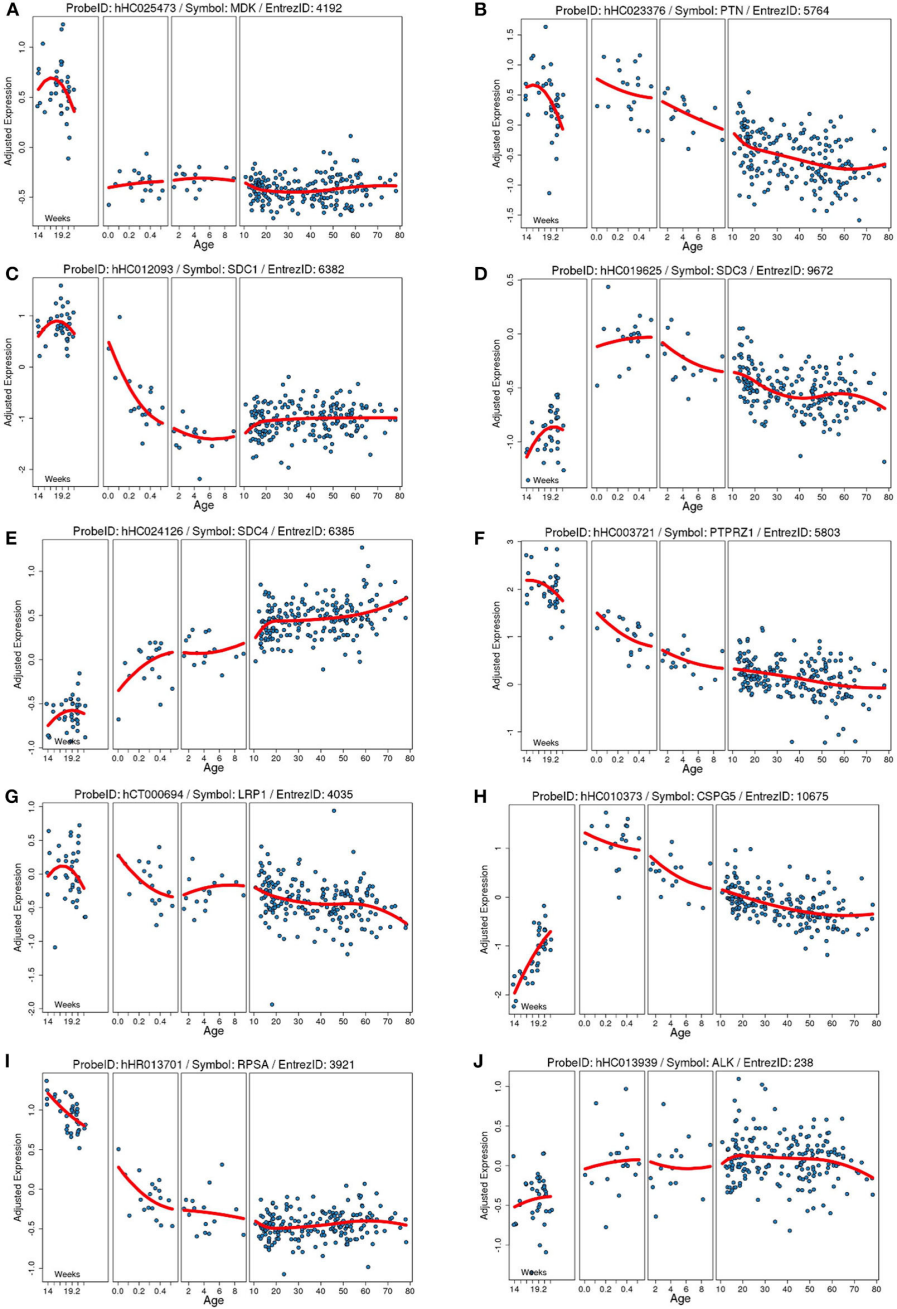
Developmental expression of human mRNA in the cortex for our proteins of interests and known receptors. Each plot shows the gene expression from a frontal cortical sample on the Y axis (age) adjusted to the total average global gene expression across timepoints. Each blue dot is an individual human sample, and the red line is the moving average of gene expression. Age on the Y axis is in weeks of gestation (far right) and then in years after birth. **(A)** Midkine (*MDK)*. **(B)** Pleiotrophin (*PTN*). **(C)** Syndecan-1 (*SDC1*). **(D)** Syndecan-3 (*SDC3*). **(E)** Syndecan-4 (*SDC4*). **(F)** Protein tyrosine phosphatase ζ (*PTPRZ1*). **(G)** Low-density lipoprotein receptor-related protein (LRP1). **(H)** Neuroglycan C/chondroitin sulfate proteoglycan 5 (*CSPG5*). **(I)** Laminin binding protein precursor/40S ribosomal protein SA (*RPSA*). **(J)** Anaplastic lymphoma kinase (*ALK*). Data collated from the http://braincloud.jhmi.edu/.

**Figure 2:**
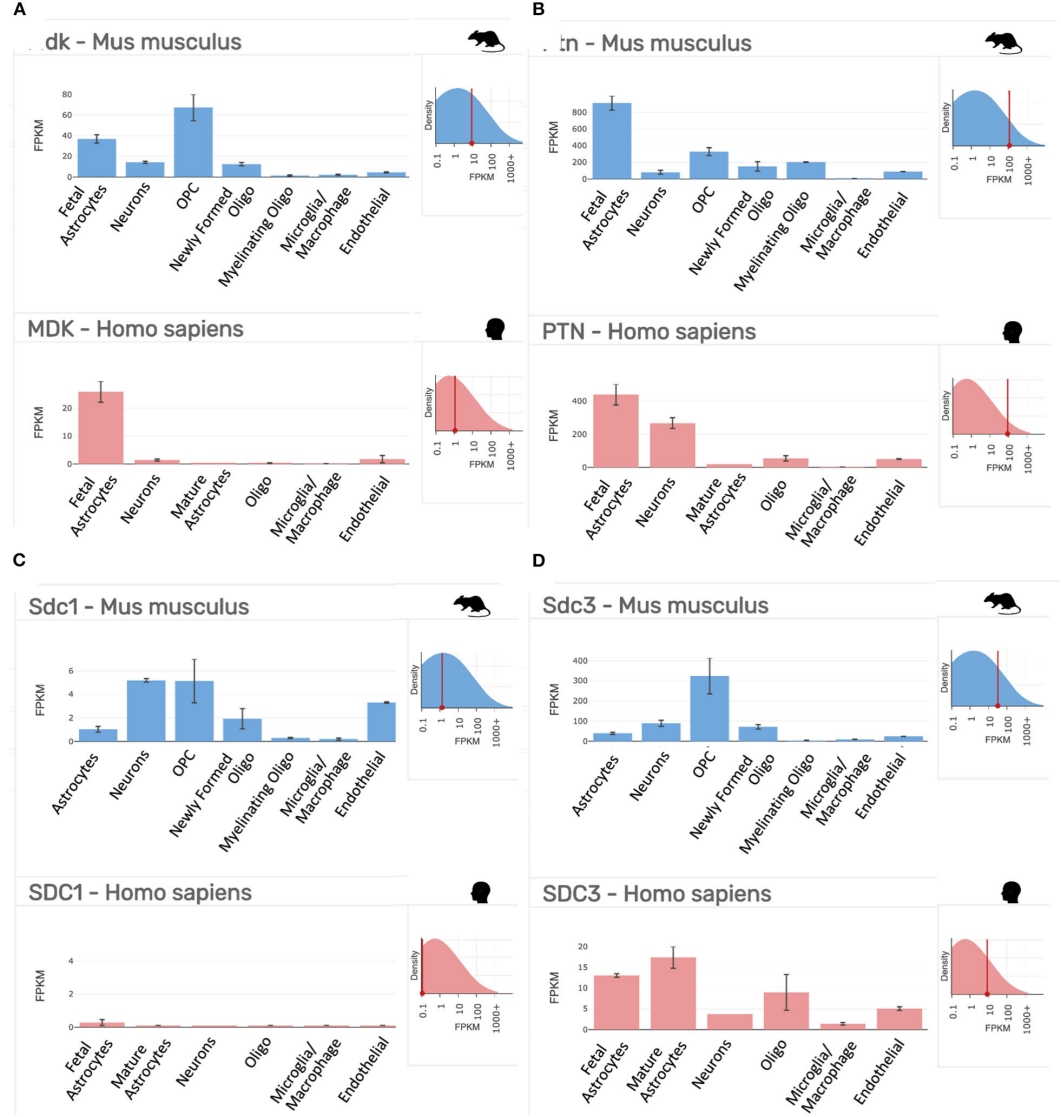
Gene expression profiling data of our targets of interest in purified cell populations from the mouse (*Mus musculus*, blue) and human (*Homo sapiens*, red) at stages of development as indicated. **(A)** Midkine (*Mdk*/*MDK)*. **(B)** Pleiotrophin (*Ptn/PTN)*. **(C)** Syndecan-1 (*Sdc1/SDC1*). **(D)** Syndecan-3 (*Sdc3/SDC3*). FPKM, fragments per kilobase of transcript per million mapped reads = the relative expression of a transcript. Top right inset on each panel shows the overall expression of the gene relative to all transcripts in the analysis. Data collated from the http://www.brainrnaseq.org/.

**Figure 3:**
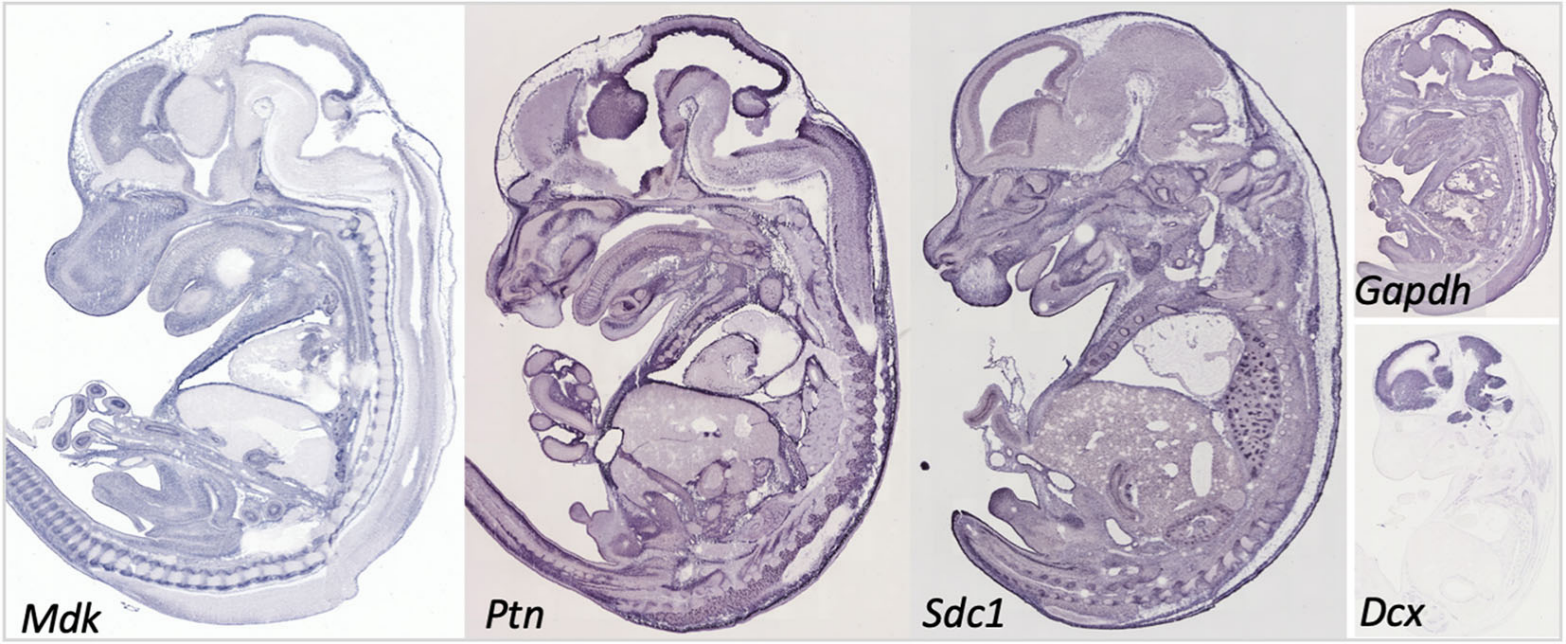
Gene expression in the embryonic day 14.5 mouse embryo for midkine (*Mdk*), pleiotrophin (*Ptn*), and syndecan-1 (*Sdc1*). Data for Syndecan-3 (*Sdc3*) not available. Data for the common reference gene glyceraldehyde 3-phosphate dehydrogenase (*Gapdh)* and the immature neuronal gene doublecortin (Dcx) shown to help understand the relative intensity in our targets. Data collated from Genepaint: https://gp3.mpg.de/.

**Figure 4:**
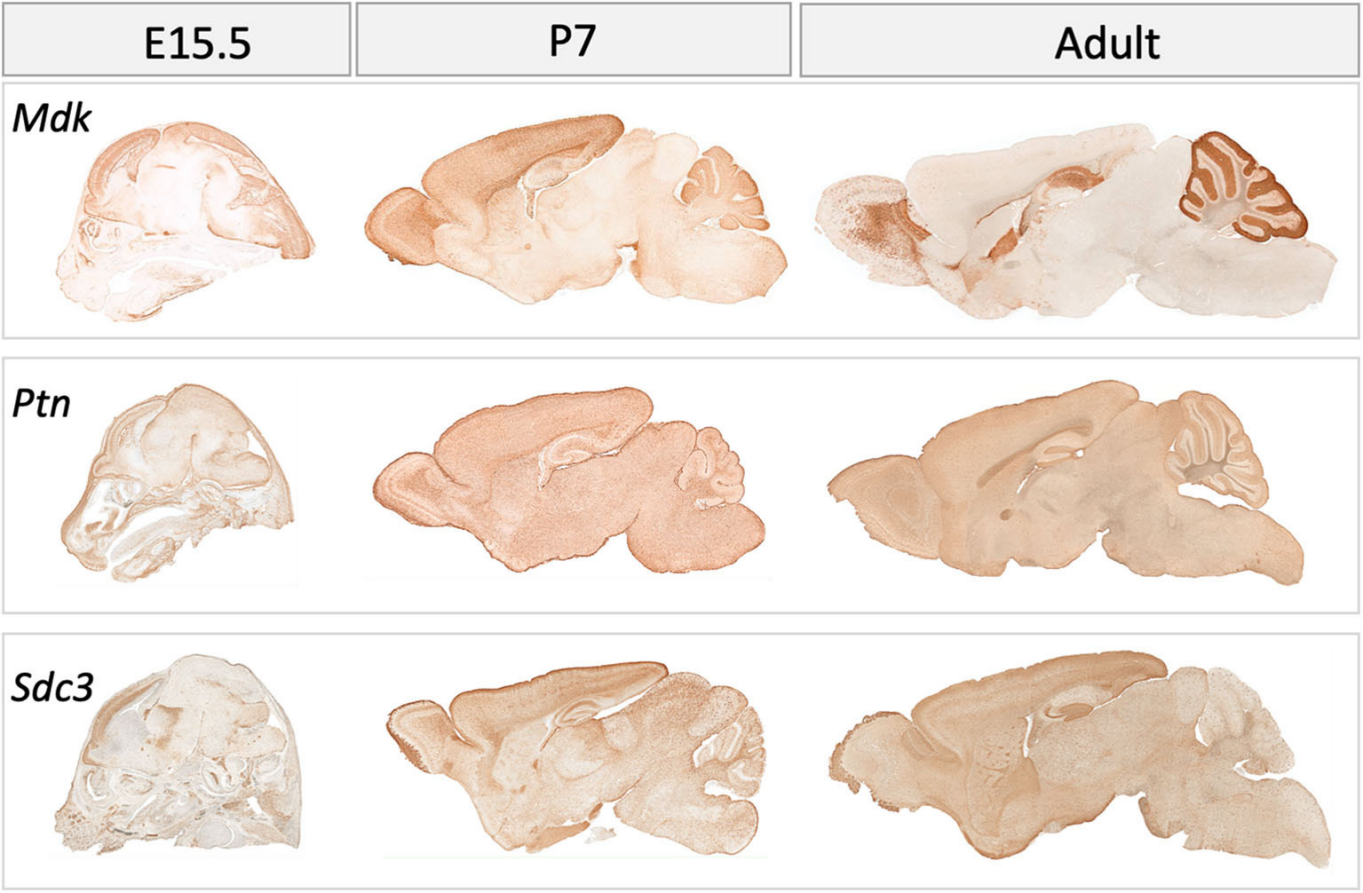
Gene expression in the mouse brain at embryonic (E) day 15.5, postnatal day (P) 7 and in adulthood for midkine (*Mdk*), pleiotrophin (*Ptn*), Syndecan-3 (*Sdc3*). Data for Syndecan-1 (*Sdc1*) not available. Data collated from GENSAT: http://www.gensat.org/index.html.

**Figure 5:**
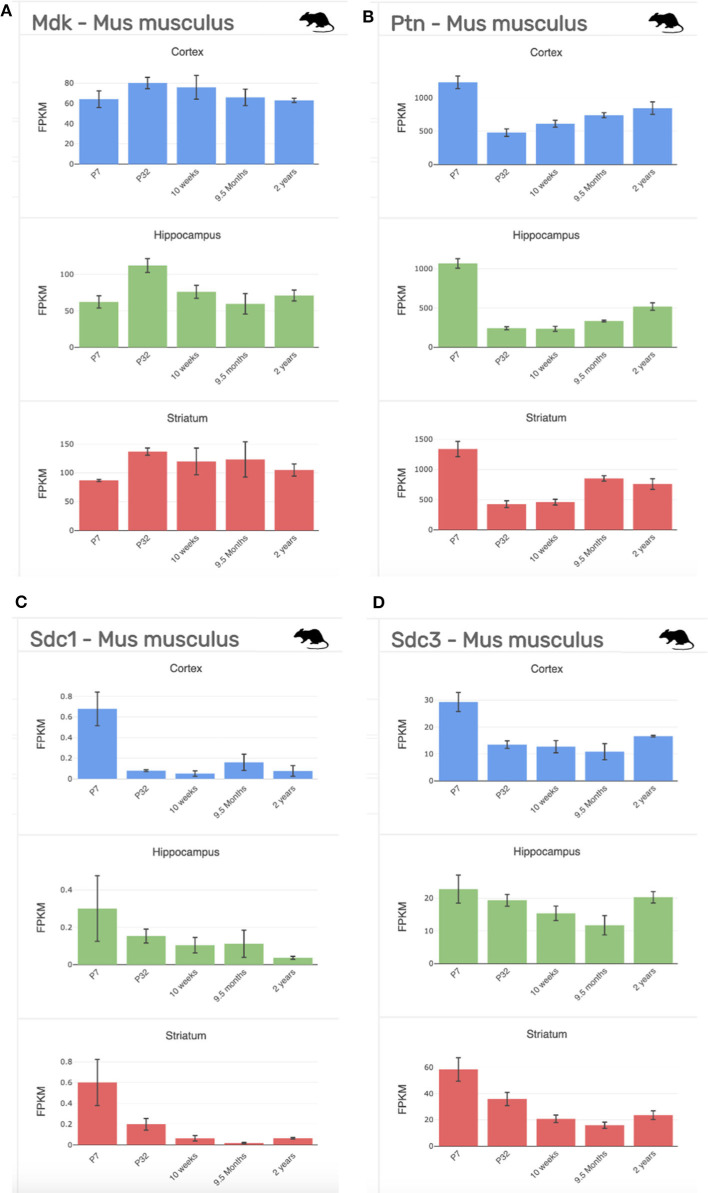
Gene expression profiling data of our targets of interest at stages across development (P7-2 years) and across regions of the brain (cortex, blue; hippocampus, green; striatum, red) from the mouse (*Mus musculus*). **(A)** Midkine (*Mdk*). **(B)** Pleiotrophin (*Ptn)*. **(C)** Syndecan-1 (*Sdc1*); **(D)**. Syndecan-3 (*Sdc3*). FPKM, fragments per kilobase of transcript per million mapped reads = the relative expression of a transcript. Data collated from http://www.brainrnaseq.org/.

MK gene expression is regulated by retinoic acid, a derivative of vitamin A (43, 44). MK's role in promoting cell proliferation, differentiation, and mitogenic senescence during development is once it shares with other *trans*-retinoic acid or retinoic-derived gene products (45, 46). The most complete understanding of the developmental expression of MK and PTN comes from zebrafish and the altricial mouse and rat. In the sections below, we outline this data and highlight what little is known for humans, while also presenting data available from public datasets, as summarized in Figures 1–**5**. In contrast to the staging of developmental events in rodent (and of course zebrafish), in humans, organogenesis occurs predominantly *in utero*. Therefore, it is subject to the influence of the maternal and intrauterine environment, and to the influence of hormones and growth factors released by the placenta (47, 48). In this regard, study of model pregnancies closer to human pregnancy might be very important. For instance, it is known that glucocorticoids negatively regulate MK expression (49). Specifically, glucocorticoids downregulate MK expression in alveolar cells isolated from fetal mouse lungs, and prolonged and exaggerated MK expression occurs in adult mice lacking the glucocorticoid receptor (49). Hence, the observation that MK decreases after mid-gestation in some species might reflect the increased fetal glucocorticoid secretion that occurs toward the end of gestation, a process required for functional maturation of many organs, including the lungs (50, 51), heart (52), and gut (53).

### Prenatal Expression

As indicated above, MK is named due to its high expression in mid-gestation. This expression pattern in humans is highlighted in Figure 1, but this data is from total cortical extracts, hiding any cell-specific variance. Cell-type specific analysis using RNA-sequencing across fetal and adult time points revealed that across cell-types, MK expression is approximately 10-fold greater in *Mus musculus* (house mouse) when compared to that of humans (Figure 2). The most complete ontogenic description of mammalian MK expression comes from rodents (summarized in Table 3), for which localization of MK and PTN proteins overlap in the embryonic stages (see also Figures 2–5). In the mouse, intense expression of MK mRNA can be detected as early as embryonic day 5 (E5) in the ectoderm and in the allantois and chorion of the placental tissues (54). Then by E8.5, MK expression is found throughout the whole mouse embryo as well as in the extra-embryonic membranes (amnion and yolk sac) (54), and in the placenta at E11.5 (55). Also, in the mouse embryo, strong MK mRNA is present throughout the developing cortical plate at E14.5 and E15.5 (Figures 3, 4), and is also present in the jaw, hindlimb bud, skin, placental capillary endothelial cells, brain, and spinal cord (44) (Figure 3).

**Table 3:** Developmental expression of MK and pleiotrophin (PTN) in the rat brain.

	**Location**	**Embryonic (E) or postnatal (P) days**	**PTN expression**	**MK expression**	**References**
Prenatal cerebral cortex	Ventricular zone	E10	⇧	⇧ ⇧	(4)
	Ventricular zone	E14	⇧ ⇧	⇧ ⇧	
	Preplate	E14	⇧	⇧ ⇧	
	Ventricular zone and intermediate zone	E17	⇧ ⇧ ⇧	⇧ ⇧ ⇧	
	Subplate	E17	⇧ ⇧	⇧ ⇧	
	Cortical plate	E17 and E18	⇧ ⇧	⇧ ⇧	
Postnatal cerebral cortex	Choroid plexus	P7	Not detected	⇧ ⇧ ⇧	(4)
	Corpus callosum	P7	⇧ ⇧ ⇧	Not detected	
	Cortical plate, marginal layer, and layer V	P1	⇧ ⇧ ⇧		
	Layer VI	P1	⇧ ⇧		
	Layers I, III, and IV	P7	⇧ ⇧ ⇧		
	Layers II, V, and VI	P7	⇧ ⇧		
	Layers I, II, III, V	P14	⇧ ⇧ ⇧		
	Layer IV and VI	P14	⇧ ⇧		
Postnatal cerebellum	PLZ, PM, ML and PCL	P1	⇧ ⇧ ⇧	⇧	(3)
	EGL, ML and PCL and WM	P3 and P5	⇧ ⇧	⇧ ⇧	
	IGL	P3 and P5	⇧	⇧	
	EGL, ML and PCL and WM	P7	⇧ ⇧ ⇧	⇧ ⇧ ⇧	
	IGL	P7	⇧	⇧	
	IGL WM	P14	⇧ ⇧ ⇧	Not detected	
	ML and PCL	P14	Not detected		

*EGL, external granular layer; IGL, internal granular layer; MK, midkine; ML, molecular layer; PTN, pleiotrophin; PMZ, pre-migratory zone; PLZ, proliferative zone; PCL, Purkinje cell layer; WM, white matter*.

In rats, MK protein immunoreactivity (IR) is found in the ventricular zone of the cerebral vesicle at E10, whereas little PTN-IR is detected here at this time. By E17 in the rat, both MK and PTN-IR emerge radially from the ventricular zone into the telencephalon, and dual expression is most intense in the intermediate zone and subventricular zones beneath the subplate. Moderate expression of MK and PTN is found within the subplate, and most importantly, coexpression in the cortical plate is localized to the radial glial processes (56)–a network that governs migration of postmitotic neurons (57). Expression patterns for MK protein at E17 (4) and MK mRNA at E14.5 and E15.5 are similar (Figures 3, 4).

Of note, in human pregnancies, MK and PTN proteins are present in amniotic fluid in normal mid-term pregnancy, during preterm labor with or without rupture of the membranes, and at term with and without labor (58); the significance is discussed further below.

### Postnatal Expression of Midkine

In postnatal life (P7), protein expression for MK in the forebrain of the rat is now largely restricted to the choroid plexus (4). However, there is an obvious discrepancy in this restricted protein expression and mRNA levels, as mRNA data shows that mRNA for MK is still robustly expressed at P7 across the cortex (Figure 4). On the other hand, PTN protein shows a distinct spatiotemporal pattern of expression during completion of cortical lamination—corresponding to the inside—out development of the cortex (Table 3) (4). Specifically, PTN-IR is localized in the cell surface of neuronal cell bodies and processes and is intensely expressed in the marginal layer at P1, and cortical layer I at P7 and P14. PTN-IR is also robust in the corpus callosum when assessed at P7, localized in the axoplasm and, to a lesser extent, the surface of callosal fibers. In the forebrain, PTN-IR intensity peaks at P7–P14, consistent with other reports of PTN mRNA peaking at around this age before decreasing progressively into adulthood in humans (Figure 1) and in rats (4, 59). Across postnatal life in rats (P7–2 years), mRNA studies indicate that the regional heterogeneity in expression of MK and its associated receptors continues (Figure 4).

In the developing rat cerebellum in early postnatal life, MK and PTN protein are coexpressed (3) (see Table 3). From P1 to P5, PTN protein expression is found in the cerebellar cortex; at P3, MK protein expression is colocalized with PTN, and MK-IR reaches similar levels to PTN by P5. Most importantly, at P7, intense expression of both proteins is colocalized to neural and glial processes extending downward from the external granular layer, through the molecular layer and Purkinje cell layer, with weaker expression in the internal granular layer but with intense expression in the white matter. This pattern of expression is associated with Bergmann glial processes (3, 60)—a cerebellum-specific radial glial network that mediates migration of postmitotic neurons (61). Expression of MK diminishes from P7 to P14, while PTN-IR becomes restricted to the internal granular layer and the white matter (4). This comprehensive study of MK and PTN protein covered the developmental ages of E17, P7, and P14, but mRNA data for MK in the adult rat cerebellum (Figure 4) illustrates a robust expression of MK transcript in the molecular layer that is worth further investigation.

### Developmental Importance of MK Expression

During embryonic and early postnatal development in these altricial rodents, both MK and PTN are highly expressed in neurites and glial cell extensions (3, 4) and are key in regulating neurite outgrowth (62–64), earning their membership in the neurite outgrowth family. Co-expression of PTN and MK in the embryonic stages of forebrain development is prominent in regions where cell migration and neurite outgrowth occurs (4), and the same is also true for the postnatal cerebellum, where MK and PTN likely combine to mediate the development of fiber networks (3).

Gene expression and protein studies suggest that MK signaling in the rat embryo occurs predominantly through members of the Syndecan (Synd) family, namely, Synd-1 and Synd-3 (35). Each of the four Synd family members have a specific, developmentally regulated pattern of tissue expression—with high expression of the Synd-1 receptor before birth, which then decreases postnatally, and high expression of the Synd-3 receptor from immediately after birth (Figures 1–3) (65). However, both receptor proteins are expressed during mid-gestation, with expression of Synd-1 at E10-12, and Synd-3 at E10-16—implicating both in the early construction of the CNS from the neural tube (66). Despite this, Synd-1 or Synd-3 knockout (KO) mice are viable and fertile (67–69). However, Synd-1 KO mice present with a growth restricted phenotype, being, on average, 15% smaller in weight throughout the first 4 months of life, and this is detected as early as E17.5 (69). In addition, Synd-3 KO mice exhibit impaired migration of glia and neurons in the cerebral cortex during development, resulting in fewer neurons residing in the superficial cortical laminae when assessed later in adult life (68). Both MK and PTN facilitate neurite outgrowth *in vitro* (43, 70), and so MK and PTN signaling via the Synd-3 receptor during corticogenesis is implicated in axonal/neural migration. Moreover, Synd-3 KO mice exhibit enhanced long-term potentiation in the CA1 region of the hippocampus, resulting in impaired memory formation as assessed using water maze and fear conditioning assays (67).

As outlined in mRNA datasets in Figure 2, Synd-3 is more highly expressed than Synd-1 in both *Mus musculus* and humans. More importantly, RNA sequencing suggests that the density of Synd-1 expression is approximately 100-fold less in humans than that of the *Mus musculus*, and this difference is 50-fold less for Synd-3. It is unknown currently if these differences are real (71), and if they are, whether there are differences in protein abundance or stability in humans that counteract any higher expression of the mouse mRNA. Irrespective, it is an example of how results from rodent experiments must be interpreted cautiously considering that this difference may indicate fundamental physiological differences between organisms that prevent simple extrapolation of findings (72).

Difficulties in understanding the specific role of MK come from the fact that MK interacts with several receptors (Table 2), each of which have numerous potential ligands, thus limiting the ability to delineate the activity of MK through receptor blockade or KO studies. Also, there are structural and functional similarities and complex feedback loops in actions between MK and PTN. These lead to redundancy, illustrated by the fact that (73), to date, there are no conditional KO mice for MK to provide more nuanced data on functions. Nevertheless, MK KO mice do still show an abnormal phenotype (74). Brain-specific structural abnormalities in MK KO studies include delayed hippocampal development, as shown by a transient abnormal increase in calretinin in the granule cell layer of the dentate gyrus (75). However, at this young adult stage of life for a rat, behavioral analysis unveiled that MK KO mice exhibit increased anxiety and impaired working memory assessed via the elevated plus maze test and y-maze test, respectively. (67), suggesting that MK activity via Synd-3 may serve to modulate hippocampal synaptic plasticity. MK KO mice also exhibit reduced striatal dopamine content, which has been interpreted as an increased vulnerability for development of behavioral disorders such as schizophrenia and autism, and abnormal serum levels of MK have been reported in people with these behaviors (76).

MK/PTN double KO (DKO) mice have been previously produced by cross breeding mice heterozygous for MK and PTN deficiency. DKO mice are born at one third the expected frequency based on Mendelian segregation (77). The authors attributed this to lethality occurring prior to E14.5; however, no histological abnormalities of organ architecture were found at this stage, and an earlier assessment was not made. DKO mice present with a severe postnatally developing growth retardation (50% reduction at 4 weeks of age) not corrected with high-calorie postnatal feeding (77). As mentioned previously, Synd-1 KO mice also present with a growth-restricted phenotype evident as early as E17.5 (69). We speculate that MK and PTN signaling via Synd-1 may be of functional importance during growth and may provide key insights for exploring interventions to ameliorate fetal growth restriction. MK/PTN DKO mice at 4 weeks of age exhibit 40–50% reduction in the spontaneous locomotor activity compared to wild-type (WT) mice, with the difference resolving somewhat to a 20% deficit at 3 months of age (77). Interestingly, MK, KO, and/or PTN KO mice have auditory deficits, but in the case of DKO, the deficit is more severe, consistent with a role for both PTN and MK in regulating the tectorial membrane proteins α- and β-tectorin that are crucial proteins for cochlear development (78).

MK and PTN proteins are present in the amniotic fluid from at least midgestation to birth in normal pregnancies and those disrupted by preterm labor (58). The functional significance and the source of these heparin-binding growth factors in amniotic fluid are unclear. It is known that both MK and PTN are expressed during development of the epithelial-mesenchymal interactions in the fetal lung (2, 49) and gastrointestinal tract (2), and MK is expressed in embryonic mouse keratinocytes in the epidermis (79). As such, MK in the amniotic fluid may be participating in, or be a by-product of, the development of the epithelia of the lung, gastrointestinal tract, and skin. Of course, fetal urine is a major contributor to amniotic fluid, and amniotic MK may also come from the fetal kidney, consistent with the observation that MK is highly and constitutively expressed in the kidney (80) and is involved in kidney nephrogenesis in the fetal rat (81). The human fetus swallows amniotic fluid regularly for at least the last half of pregnancy, and as such, amniotic MK may have a role in promoting development of the luminal epithelium of the gastrointestinal tract. Finally, given the current interest in the use of amniotic stem cells for regenerative repair of perinatal brain damage (82), it is worth investigating if amniotic MK enhances the survival and potential for further differentiation of these cells, as already shown for neurogenic stem cells *ex vivo* (58, 83).

## Neuroprotection and Repair

**Figure 6:**
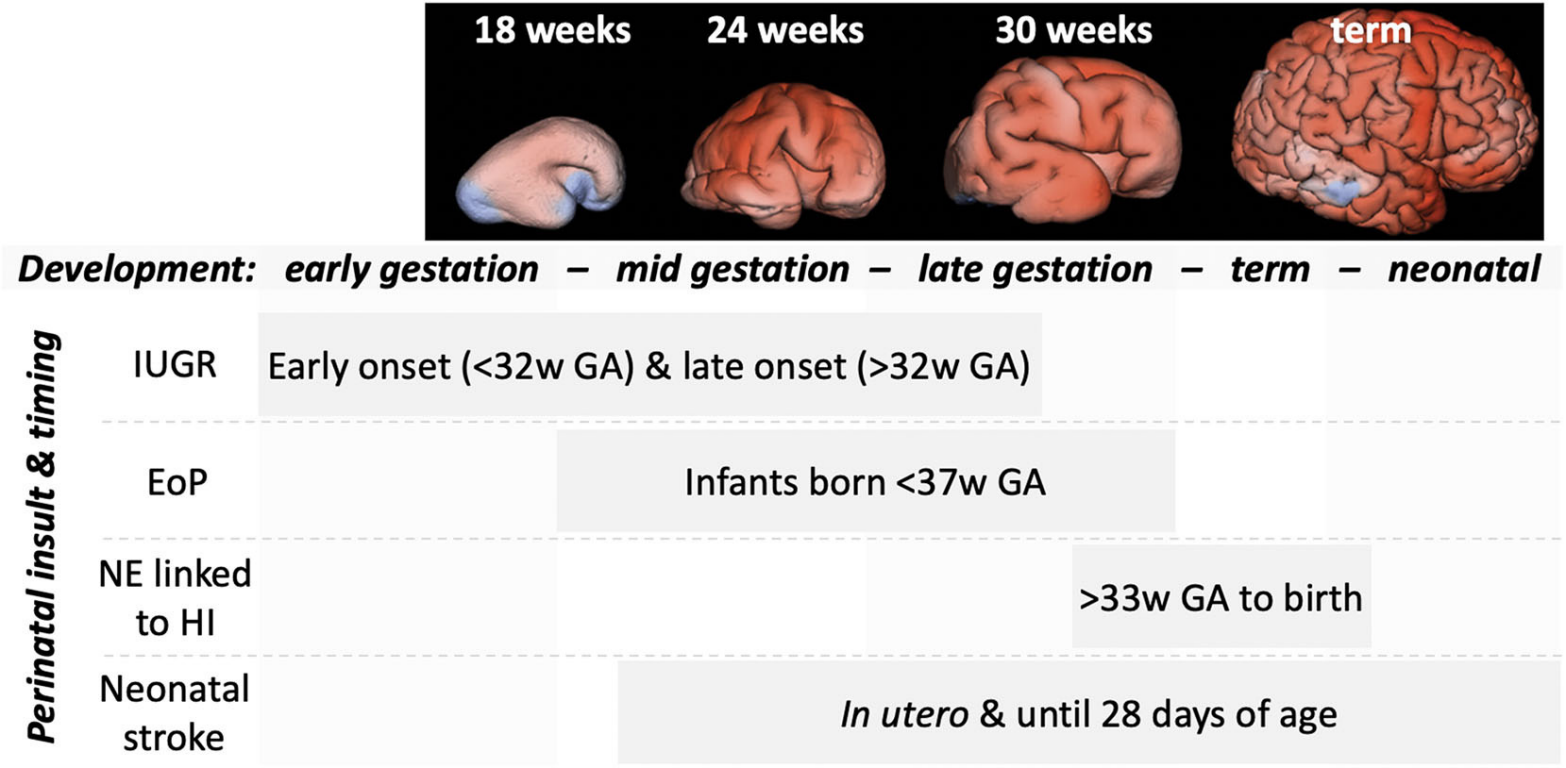
An outline of the timing of the more common perinatal insults relative to gross brain development. MRI panels adapted from Vasung et al. (90). Top, representative MRI reconstructions showing the striking changes in brain development across the weeks of gestational development. Bottom, indications of the timing (weeks of gestational age) of common perinatal insults.

We outline in this section, the primary neuropathological processes occurring in perinatal brain injuries and how these may be influenced by MK. A summary of these processes for encephalopathy of prematurity (EoP) and NE linked to HI is given in Table 4 together with a summary of proposed MK approaches. The focus of this review is perinatal brain injury, but it is highly likely that MK would also be useful for targeting damaging processes occurring during adult brain injury, such as stroke or traumatic brain injury (8, 87). Injury process do differ by age though, for example, that cell death in the developing brain is more likely to occur via a caspase-dependent process (88) and that microglia are in a “brain building” mode in the developing brain, compared to an adult homeostatic role (89). We wish to highlight that the developmental window in which a perinatal injury occurs is a key determiner of the type of damage that is caused (see Figure 6). For example, in preterm born infants, there is a widespread interneuronopathy as these cells are still migrating throughout the last trimester, a period of development disturbed by their early birth (91, 92). Interneuron damage in neonatal stroke and HIE is limited to the region of frank cell loss (93, 94). A role for HIE in preterm born infants is not conclusively supported (95), but oxygen variability is a likely contributor to injury (96). Also, myelination deficits are predominantly localized in areas such as the posterior limb of the internal capsule and the corona radiata in infants with HIE (97, 98). However, in infants with EoP, white matter injury is diffuse due to the vulnerability of populations of oligodendrocytes across the brain, and this leads to global connectivity deficits (99). Other key factors governing the specific pathogenesis include insult severity, patient population (ethnicity, social demographics), and standards of care/availability of care.

**Table 4:** An outline of the common neuropathological processes occurring in babies born preterm (with EoP) and those born at term with NE linked to HI (HIE) [as reviewed by (84–86)] and the current available therapies (18–20) and proposed application of MK.

**Neuropathology**	**EoP**	**NE linked to HI**	**Potential target for Midkine**
Microgliosis	Yes	Yes	Yes
Astrogliosis	Yes, when birth <23 weeks of GA	Yes	Yes
Neuronal death	Limited	Yes	Yes
Oligo. death	In severe cases	Yes	Yes
Oligo. dysmaturation	Yes	No	Yes
Interneuron dysmaturation	Yes	No	Possibly
**Treatments**			**Potential midkine therapy**
Description	Magnesium sulfate	Hypothermia	Midkine
Timing/route	At least 1 h before birth, IV	72 h continuous cooling (32–33°C) initiated within 6 h of birth	Acute IV boli, and/or chronic intranasal
Efficacy (NNT)	No change in mortality, decreased CP (74)	Reduced mortality and rates of NDD (7)	To be determined

*EoP, encephalopathy of prematurity; NE, neonatal encephalopathy; HI, hypoxia-ischemia; GA, gestational age; Oligo, oligodendrocyte; CP, cerebral palsy; IV, intravenous; NNT, number to treat; NDD, neurodevelopmental disability*.

### MK Expression Following Injury

Following hypoxic and/or inflammatory injury, there is induction in MK expression. Specifically, MK has been shown to mediate response to injury by suppressing programmed cell death (apoptosis), modulating the glial response, propagating peripheral immune cell recruitment, stimulating angiogenesis, and enhancing proliferation and migration of neural stem cells—all of which will be discussed further below.

The MK gene harbors response elements specific to signals arising from hypoxia and inflammation (6, 40), two conditions that are frequently associated with perinatal brain damage. The hypoxia response element in the promotor region of MK (6) binds with the transcription factor, hypoxia-inducible factor 1α (HIF-1α). MK itself also increases HIF-1α expression creating a positive feedback loop for signal propagation (6). Hypoxic upregulation of MK expression likely explains the MK expression observed in tumors, where higher levels correlate with a worse prognosis (100, 101). The MK promoter region also possesses a putative nuclear factor kappa light-chain enhancer of activated B cells (NFκB) response element (7, 40). Stimuli that induce NFκB activation include reactive oxygen species, proinflammatory cytokines such as tumor necrosis factor α (TNFα) and interleukin-1β (IL-1β), and the bacterial endotoxin, lipopolysaccharide (LPS) (102–105). MK expression can be upregulated during many forms of inflammatory reaction (102–105). Consistent with this, an elegant study that used a prostate adenocarcinoma cell line showed that induction of MK expression following TNFα exposure occurs in an NFκB-dependent manner (7).

### Cell Death

Many studies demonstrate that MK is involved in the suppression of apoptosis (9, 106–108). For instance, MK treatment in serum-starved cortical neuronal cultures from E17 rats attenuates expression of caspase-3 via rapid activation of the ERK and Akt pathways by MK (106). This finding is consistent with other *in vivo* evidence that MK has an antiapoptotic effect by downregulating caspase-3 in hepatocellular carcinoma and human meningiomas (107, 108). Furthermore, *in vitro* knockdown of the MK gene using small interfering RNA (siRNA) in gastric cancer cell lines inhibits cell growth, upregulates proapoptotic Bax, downregulates antiapoptotic Bcl-2, increases activity of caspases-3, -8, and -9, and increases release of mitochondrial cytochrome c (109). Cell death occurs in the perinatal brain in response to insults including HI in term infants, and it is often ascribed to apoptotic processes (110, 111), and MK may act to ameliorate these events. However, it is not known if MK interacts with the more recently identified necroptotic pathway—a form of cell death that is initiated using the signal transduction mechanisms of apoptosis, but which culminates with a necrotic phenotype (112, 113). Necroptosis is largely mediated via receptor-interaction protein-1 (113) and is a primary contributor to cell death where it is occurring following perinatal brain injury (114–116). As such, it is now important to assess the potential involvement of MK in necroptosis.

### Reactive Gliosis

Microglial activation is almost ubiquitously reported across forms of perinatal brain injury, and a causal role for these processes in injury has been demonstrated across paradigms (117, 118). Conversely, a neuroprotective role for microglia is also shown in other experimental settings (119, 120). It is now generally regarded that microglia play numerous different roles in brain injury, dependent on the nature, severity, and stage of development of the injury, and the timepoint of the analysis (121, 122). Analysis of microglia in MK KO mice has found no significant effects on distribution and morphology in adulthood before or after LPS challenge (123). Another study used fluorescently activated cell sorting (FACS) to phenotype microglia from these mice and observed that MK^−/−^ microglia had higher expression of B7-2 (cluster of differentiation-80), macrophage chemotactic protein-1 (MCP-1), and IL-1β, and these differences were lost once the cells were activated with either LPS or interferon γ (IFNγ) (124).

A pathological role for astrocyte activation becomes apparent in infants older than 28 weeks gestational age, linked to maturation of these supportive glia at that time (125–127). These cells increase production of proinflammatory cytokines and proliferate around focal lesions sites (128, 129). As such, activation of astrocytes is reported in post-mortem studies of infants with encephalopathy of prematurity (126, 130) and infants with NE linked to HI (127, 131). As for microglia, astrocytes also play roles in protecting the brain, with injury leading to increased uptake of glutamate, production of antioxidants, and production of trophic factors [as reviewed by (132)]. Animal studies of brain injury, such as transient cerebral ischemia causing damage to the hippocampal CA1 region in adult male rats (133), and kainic acid-induced epileptic seizure injuries in adult male mice (9), show that reactive astrocytes produce MK. Similarly, astrocytes in autopsied adult human brains express MK 4 days after ischemia (134), and MK is produced by fetal human astrocytes in culture (135). Furthermore, following middle cerebral artery occlusion (stroke model) in the rat, MK expression is induced in the reactive astrocytes localized to the zone surrounding the ischemic tissue at 4 to 14 days following stroke (136). Glial-fibrillary acidic protein (GFAP)-IR astrocytes are not only a source of MK but also produce chondroitin sulfate proteoglycans (CSPGs)—broadly considered to impede neuronal regeneration (137). In a rat model of spinal cord injury, MK treatment overrides the inhibitory effects of CSPG's on neurite outgrowth (138). Specifically, MK treatment significantly improved functional recovery of injured rats as assessed using open field locomotor performance, grip strength, and correct paw placement (138). Importantly, exogenous MK treatment in *in vitro* purified astrocyte and microglia cell cultures did not induce reactive astrocytosis or microgliosis (138).

Adding complexity to the role of MK in modulating reactive gliosis is evidence of the response being stimulus dependent and region specific. For example, amphetamine-induced reactive gliosis (GFAP-IR) is enhanced in the striatum of adult MK KO mice compared to WT; however, hippocampal GFAP-IR is reduced (139). In contrast, adult MK KO mice treated with LPS have reduced striatal GFAP-IR compared to WT—and in the prefrontal cortex, there is a decrease in the perimeter and circularity index of ionized calcium binding adaptor molecule 1 (Iba1; marker of microglia/macrophage) cells in MK KO compared to WT (123). Of interest is that MK enhances migration of microglia/macrophage isolated from the forebrain of newborn mice when assessed *in vitro* (140). As such, MK may contribute to the recruitment of microglia following injury to the developing CNS.

### Oligodendrocytes and Myelination

Oligodendrocyte maturation occurs via a highly orchestrated set of sequential processes for which many of the numerous regulatory pathways are known (141). How oligodendrocytes are affected in forms of perinatal brain injuries depends on the timing, nature, and severity of the brain injury (142–145). Very generally, research in post-mortem and preclinical models illustrates that severe injury at any time of development can lead to the death of oligodendrocytes (142). Given the antiapoptotic abilities of MK (outlined above), it may be a valid therapy for this negative outcome of perinatal brain injury. In the preterm brain, where injury is severe enough to cause cell loss, there appears to be a compensatory proliferation, but the newly born oligodendrocyte cells then fail to mature (146). In the cases of moderate/mild injury in the preterm born equivalent brain, this oligodendrocyte dysmaturation occurs without any appreciable preceding cell death (126, 147). Moderate levels of injury are the most prevalent and only 5% are severe, cystic cases (148).

Receptors for MK (listed in Table 2) are found on oligodendrocytes, with complex interactions with other maturation pathways. For instance, Fyn tyrosine kinase-mediated downregulation of Rho activity through activation of p190RhoGAP is crucial for oligodendrocyte differentiation and myelination. MK binds to the extracellular region of the protein tyrosine phosphatase receptor type β/ζ (PTPRZ) that is highly expressed by oligodendrocyte progenitors. Interestingly, p190RhoGAP is also a substrate for PTPRZ indicating that the presence of MK can influence the activity of the p190RhoGAP cascade and, as such, oligodendrocyte maturation. It is worth noting that in the adult brain, there is a pool of oligodendrocyte precursors (OPC) responsible for the homeostatic replacement of mature oligodendrocytes (149) and (attempted) replacement of cells in the case of injury (150). PTN, via its inhibitory actions on PTPRZ, supports this homeostatic self-renewal of the OPC pool (151). In oligodendrocytes, PTPRZ signaling has antagonistic roles for PTN and MK, but it is not yet known if MK plays a role in the maintenance of this OPC pool.

There is little work on the effects of MK on oligodendrocytes directly. Preliminary reports indicate that MK exposure increases oligodendrocyte maturation in an immortalized precursor population (OL1 cells) (152). It has similarly been shown that in the oligodendroglia precursor cell line (CG4), MK acted via the neuroglycan C receptor to stimulate process extension (37). Of note, the authors also overexpressed the neuroglycan C receptor in neuroblastoma cells and were able to induce a similar MK-dependent cytoskeletal arrangement, indicating that this is not necessarily a cell type-specific effect.

Looking for further evidence for a role of MK in oligodendrocyte biology, we note that MK mRNA is increased in the demyelinated white matter of the lumber spinal cord in a mouse model of myelin oligodendrocyte glycoprotein-induced experimental autoimmune encephalopathy (EAE) (153). Subsequent studies of MK's role in EAE (124, 154), including experimental MK therapies, have not reported specific data on the basal or post-insult state of the oligodendrocytes or myelin. However, they have reported a causal link between MK and suppression of regulatory T-cell (Treg) proliferation. We assume that these studies had explored, but had not reported, on the “low hanging fruit” of direct effects of MK on oligodendrocytes or myelin in EAE. So then perhaps the lack of information on any effect of MK on oligodendrocytes in such obvious models itself rules out a significant interaction.

### Recruitment of Peripheral Immune Cells

In adult animal models of brain injury, MK facilitates the migration of leukocytes to the site of injury, namely, neutrophils and monocytes/macrophages (12, 140). These immune cells are implicated in modulating repair pathways and positive processes such as angiogenesis (155–157). However, an excessive stimulation of these processes is known to exacerbate brain injury (118, 126, 158–160). In models of perinatal brain damage linked to stroke and moderate systemic inflammation, the role of macrophages is minimal (161, 162), but more significant in models involving HI (160, 163). Under hypoxic conditions, both neutrophils and monocytes isolated from adult human blood transiently express MK, peaking after 4 h of exposure to 1% oxygen (11), and MK-IR is localized to both the cell surface and intracellular cytoplasmic vesicles. Following 6 h of hypoxia, MK expression in neutrophils decreases to near baseline levels. After 20 h of hypoxia, Western blotting techniques revealed that MK expression in monocytes resembles that of normoxic conditions; however, immunostaining for MK showed that the cell membrane is almost completely saturated in MK. However, MK is not detected in the supernatant of neutrophils and monocytes, and so it is assumed that it is not secreted from these cells. Instead, MK may participate in autocrine or cell–cell contact-dependent paracrine signaling pathways. An alternate hypothesis (proposed by 11) is that MK is internalized via endocytosis to prevent an excessive inflammatory response, as for IL-1β and the expression of its decoy receptor, IL-1RII.

MK is involved in extravasation of neutrophils. In neutrophils isolated from adult human blood, MK mediates neutrophil extravasation via interaction with low-density lipoprotein receptor-1 (LRP-1), which, in turn, causes conformational changes in β2 integrins promoting adhesion (12). It is unknown if this mechanism of extravasation is age dependent; however, neutrophil extravasation is impaired in MK KO mice *in vivo* following hindlimb ischemia, and blockade of LRP-1 impairs MK binding to neutrophils *in vitro*–suggesting an important role for MK in neutrophil trafficking. Systemic neutrophil responses are elevated in preterm born infants with brain injury (164), but it is not clear if neutrophils have a significant role in the development of brain injury in these infants (162, 165). In term-born brain injury related to HI, neutrophils are clearly involved in the sequence of events as the injury evolves (166–168), and if one effect of MK was to reduce their transmigration, this could be of significant therapeutic benefit.

The chemoattractant properties of MK for recruitment of peripheral immune cells may explain, in part, the results of a recent study on the examined traumatic brain injury (TBI) in MK KO adult mice (87). Here, MK KO mice had reduced levels of apoptosis at 7 days post-TBI as assessed via cleaved caspase 3 expression and showed improved neurological outcomes at 14 days following TBI. Importantly, MK KO mice had reduced microglial/macrophage Iba1-IR at 3 days post-TBI when compared to WT. Using immunohistochemical markers for the proinflammatory-like or M1 (CD16/32+) and anti-inflammatory-like or M2 (arginase1+) phenotypes at 3 days post-TBI, MK KO mice had fewer M1 CD16/32+ cells in the perilesional site. Furthermore, mRNA levels of other M1 markers (e.g., TNFα, CD11b) were reduced in MK KO compared to WT. Flow cytometry was used to segregate macrophages and microglia, and at 3 days post-TBI, MK KO mice had increased levels of M2 arginase1+ microglia and M2 CD163+ macrophages, but fewer M2 arginase1 + macrophages. Thus, these results suggest that MK modulates neuroinflammation by influencing the polarization of microglia toward the M2 phenotype. However, in the case of TBI, blood–brain barrier (BBB) permeability is significantly increased with effects seen up to 7 days post injury, facilitating leukocyte trafficking to the brain parenchyma (169). As such, the chemotactic properties of MK may potentiate injury to the adult CNS via amplifying recruitment of peripheral immune cells, and this could explain the reported beneficial effect of MK KO in the adult mouse model of TBI (87). Due to known age-dependent differences in BBB permeability, chemokine function, and leukocyte recruitment following CNS injury (170), it will be important to characterize the effect of MK activity in the context of the developing CNS.

Mast cells are first responders in the response of the brain to experimental HI (171, 172). There is a well-known role for mast cells in adult TBI (173, 174); however, the use of cromoglycate in a P14 rat model has shown that they appear to have no effect on injury outcome (175). A role for these cells in the less developed (preterm) brain, possibly via their lesser known homeostatic secretory roles, is unknown. It is logical that MK may have an impact on developing brain injury as it has striking effects on mast cell activation causing rapid and dose-dependent degranulation (176). Mast cells themselves produce MK, and this expression is increased in people with cystic fibrosis, which is linked to the role of MK as a host defense protein (177).

Finally, MK suppresses Treg proliferation by suppressing the activity of tolerogenic dendritic cells (124, 154). To the best of our knowledge, the role of tolerogenic dendritic cells in perinatal brain injuries is not known. Together with the host of effects on other immune cells, MK may influence perinatal brain injury via these populations.

### Angiogenesis

Following HI brain injury in the adult human, a robust angiogenic response occurs within 3–4 days (178). However, several studies indicate that in the neonate, this response is limited and occurs much later, and ischemic tissue presents with severe and chronic signs of vascular degeneration spreading beyond the site of injury (179–181). Indeed, a small study has associated the presence of proangiogenic factors in the serum of asphyxiated neonates with better outcomes (181), and although MK does not appear to be critical for the development of the vascular system (11), MK does promote angiogenesis in certain situations. Angiogenesis is severely compromised in MK KO adult mice subjected to occlusion of the right femoral artery (hindlimb ischemia model) as assessed via immunohistochemistry using markers for proliferation (Ki67) and endothelial cells (CD31) (11). Also, treatment with an antisense oligonucleotide targeting MK impairs angiogenesis in the chick chorioallantoic membrane, and reduces tumor progression *in situ* in hepatocellular carcinoma xenografts in mice (182). Indeed, Synd-1 has been implicated in regulating angiogenesis via activation of α_v_β_3_ and α_v_β_5_ integrins in human vascular endothelial cells *in vitro*, and in mouse mammary tumors *in vivo*, suggesting that activity of MK via Synd-1 may modulate angiogenesis (183). Promoting angiogenesis supports neural regeneration in a model of neonatal stroke (184), and thus, the angiogenic potential of MK following perinatal brain damage may be relevant for exploring strategies to enhance this response.

Notably, endothelial cells are thought to be a major source of soluble MK (11). MK is localized in the Golgi apparatus of human umbilical vein endothelial cells (HUVECs) following 4 h of hypoxia (1% oxygen), and the supernatant of cultured HUVECs contains increased MK levels after 4 and 20 h of hypoxia. Interestingly, intraperitoneal injection of hypoxia-preconditioned HUVECs in a neonatal rat model of HIE ameliorates neuronal apoptosis, stimulates angiogenesis, and attenuates neurovascular damage in the acute and subacute stages of brain injury—and also improves motor and neuropathological outcomes assessed later in adult life (185). The possibility that the beneficial effects of hypoxia-preconditioned HUVEC treatment may be attributable to the actions of MK is worth further investigation.

As mentioned previously, MK facilitates migration and extravasation of neutrophils and macrophages to sites of tissue injury, and these cells promote angiogenesis (186). Thus, MK's angiogenic role may arise as a result of two primary mechanisms: (i) directly by enhancing growth and proliferation of endothelial cells and (ii) indirectly by recruiting neutrophils and macrophages. With regard to the latter, resident brain microglia/macrophages dominate the site of injury in the acute stages following neonatal HI injury in the neonatal rat (161). Also, microglia have been shown to prevent hemorrhage following focal neonatal stroke in rat pups by modulating the neurovascular response (119). As such, in the context of perinatal brain injury, MK may not only enhance angiogenesis but may also facilitate migration of microglia to the damaged vasculature to preserve BBB integrity. Further work is needed to clarify this.

### Proliferation and Migration of Stem Cells and Regeneration

Inflammation and hypoxia have the effect of suppressing neuroglial proliferation in the developing brain (187, 188), although specific effects are linked to the severity of HI [as discussed by (189)]. One important effect of MK is that it promotes neural stem cell proliferation and migration, and MK is strongly expressed in migrating neurons and radial glial processes during development of the cerebral cortex and cerebellum in the rat (4). Injection of MK mRNA into the embryos of zebrafish promotes neurogenesis (190), and expression of MK in neural precursor cells in mice promotes their survival and proliferation (191). Viral-mediated short hairpin (sh) RNA knockdown of MK also significantly reduces sympathetic neuron proliferation (42), where MK-mediated proliferation was shown to occur largely through the receptor anaplastic lymphoma kinase (ALK), as shALK and shMK treatment in sympathetic ganglia resulted in a similar reduction in proliferation (42). In zebrafish, the *ALK* ortholog *leukocyte tyrosine kinase* (*ltk)* is critical for neurogenesis of the developing CNS, where its overexpression increases proliferation of neural progenitors (192). Indeed, MK-mediated cell proliferation via ALK activation has been implicated in many cell types and occurs via activation of downstream signal transduction pathways PI3K and MAPK (41).

Unlike mammals, zebrafish can regenerate photoreceptor neurons (193), providing a platform to assess MK's role in neuronal regeneration. The zebrafish has two species-specific MK genes—*mka* and *mkb* (194). Analysis of retinal development and regeneration in zebrafish identified that *mka* is highly expressed in stem cells destined to be retinal horizontal cells, and in the outer layer of the retina, a prominent site of stem cell differentiation (195). Importantly, *mka* is transiently expressed in Müller cells in the developing retina—a retina-specific radial glial network (195). Conversely, the zebrafish *mkb* is transcribed in newly postmitotic cells deeply entrenched in the inner retinal layers, and in the amacrine cells and other components in the ganglion cell layer (195). During retinal development, MKA protein is localized in the neuroepithelium and the retinal margin and remains localized in the circumferential marginal zone of the retina (196). Following photolytic death of photoreceptors, *mka* adopts a center-to-peripheral pattern of expression in rods and cones as photoreceptor function regenerates (196). Thus, MK may be a trophic factor critical for development of the retina that also mediates regenerative processes. The developing eye is very sensitive to hypoxia and hyperoxia (197, 198)—consistent with reports of retinal atrophy in human infants following perinatal asphyxia (197) and “retinopathy of prematurity” that occurs due to supraphysiological levels of oxygen (198, 199) and exposure to perinatal inflammation (200). Currently, there is no effective treatment to specifically repair the infant's retina from such damage, and MK may provide the effective therapy needed here. This is further supported by research that showed that intravitreal injection of MK rescued retinal damage induced by exposure to high intensity light in adult rats (201).

In a rat focal brain ischemia model utilizing photoembolism, MK overexpression produced by ipsilateral injection of an adenoviral vector into the lateral ventricle 90 min after injury resulted in proliferation of neuronal stem cells, reduction of infarct volume by ~33%, and an increase in the number of callosal and subventricular neuronal cells expressing other migratory factors (202). These neuroprotective effects of MK gene transfer persisted beyond the acute phase of infarction. Taken together, there is evidence to support MK's therapeutic potential in enhancing neuronal regeneration following injury to the CNS.

## Challenges and Potential of MK Treatment in the Perinatal Period

As discussed above, MK has been shown to have diverse actions—in a cell type and developmentally regulated manner. These include effects on neurogenesis, neuronal survival, apoptosis, and glial activation across inflammatory and hypoxic CNS insults. There is an obvious promise for the use of MK in neonates with perinatal brain damage, but careful consideration needs to be made to factors such as patient selection (i.e., HIE with or without exposure to additional inflammatory challenge (203), the mode of MK delivery (systemic, intranasal, intracerebroventricular), and the timing to address the acute, secondary, or tertiary phases of injury (204).

Systemic delivery of MK is appealing because alongside the brain, other organs susceptible to inflammatory and hypoxic damage in preterm and term neonates include the heart, lung, kidney, and skeletal muscles, and in particular the diaphragm [reviewed by (205)]. In term-born infants, a focus was on understanding injury to the brain, but it is important to note that NE can develop secondary to cardiac, pulmonary, hepatic, and renal dysfunction, and is often exacerbated by systemic and cerebral inflammation (206, 207). While brain-specific treatment is an obvious clinical goal, there is the need to protect other vital organs, and it is here that MK might offer benefits in addition to the rescue of brain damage. For example, MK has been shown to be cardioprotective in rodent (208), rabbit (209), and pig (210) models of ischemic myocardial infarct by virtue of both its antiapoptotic and potent angiogenic actions, leading to reduced infarct size, less left ventricular scarring, improved cardiac performance, and overall survival (211). Importantly, the intracardiac injection of MK protein ameliorated heart failure not only when delivered at the time of cardiac ischemia/reperfusion injury but also following a delay of up to 14 days post-infarct (208). This important finding suggests that MK treatment might benefit neonates when given at various times after birth. That is, unlike therapeutic hypothermia where the “window of opportunity” to have an impact on perinatal HIE is only hours after birth (17, 212, 213), MK treatment might not be so time critical. It may be with caution that trials of systemic MK are undertaken though, given the plethora of diverse effects of MK on immune cells that may conflict with supportive recovery and overall health of the infant. The complexity of cross-organ effects is shown in studies where a putative therapy, erythropoietin, used to prevent brain damage in preterm born babies (214–216) has damaging effects on the lung in preterm lambs (217, 218). In the case of term born infants, it is also worth noting that therapies need to be tested in relation to hypothermia, the current standard of care. In this case, careful pharmacokinetics need to be assessed as it has recently been shown that cooling can lead to toxic bioaccumulation of potential neurotherapeutics (219).

The BBB in the healthy neonate is functional and more than capable of excluding circulating factors, as reviewed elsewhere (220). There is no specific evidence of the ability (or not) of MK to cross the BBB, although many neurotrophic factors are known to not penetrate the brain parenchyma sufficiently to facilitate neuroprotection (221). Evidence indicates that many forms of perinatal brain injury open the BBB to varying degrees related to the severity of the insult (162, 222–224). This regionally selective opening of the BBB may then be a useful strategy to deliver MK to the sites of injury while keeping it away from the uninjured parts of the brain. Even so, the many pro- and anti-inflammatory systemic effects of MK might mitigate against it as an effective therapeutic agent but highlights the need for more experimental evidence.

The poor penetration of MK into the brain could be assumed to be reflected in the many experimental studies that use delivery of MK by intracerebroventricular or direct intracranial injection (9). Obviously, this delivery route comes with substantial risks in infants, but it is worth noting that MK administration may be no more complicated than the surgical techniques developed for treating intraventricular hemorrhage or hydrocephalus. For example, the DRIFT procedure, necessitating the placement of two shunts into the infant's brain (225), has been able to improve the cognitive quotient of children with severe intraventricular hemorrhage by as much as 23 points (226). So, although not ideal, as the future of MK testing proceeds, it may be worth including intracerebroventricular delivery as it might directly target brain injury and avoid the side effects of systemic administration.

Two other approaches for MK delivery are of more obvious value for newborn infants. First, the intranasal route, which has been used to deliver diverse proteins to the brains of neonatal and adult experimental animals (e.g., insulin, IGF1, Fgf2, C3a, EGF, anti-tPA, osteopontin) for protection and repair (104, 227–230). What is lacking here is the experimental data on the distribution of MK after nasal delivery. Second, a route in might be mediated by new nanoparticle. For instance, a polyamidoamine dendrimer has been used to successfully deliver n-acetylcysteine to reduce brain injury in a rabbit model of cerebral palsy induced by hypoxia/ischemia (231, 232). In addition, a poly(lactic-co-glycolic acid)-poly(ethylene glycol) (PLGA-PEG) nanoparticle has successfully increased the delivery of the histone deacetylase inhibitor, curcumin, to the brain in a model of term infant NE related to HI (233). An interesting biomaterials approach involves the engineering of nanoparticles to release their “cargo” in response to tissue damage–in this case–in response to acidosis in the brain subsequent to ischemia (234). This approach is valuable because it reduces possible off-target effects of drug delivery. Indeed, highly targeted effects are possible, as shown by Van Steenwinckel et al. who showed that 3DNA nanocarriers administered intraperitoneally not only cross the BBB but are taken up specifically by microglia and not by liver, bone marrow, or spleen macrophages (118). This enables this 3DNA nanoparticles to target peptides, small molecules, siRNA, and microRNA, and as such, could be used to deliver MK itself or an MK modulator. 3DNA nanoparticles are 200-nm diameter constructs that escape the endosome to enable intracellular delivery of the tagged “cargo,” and appear to have no toxicity *in vitro* or *in vivo* (235), and can be tagged with fluorophores for tracking—making them an exciting prospect for future studies.

## Conclusion

Current methods of preventing or treating perinatal brain damage have low success rates, and as such, there is a need to develop novel therapeutic approaches. The evidence summarized here shows the potential for MK, as an endogenous growth factor and cytokine, to be used to mitigate processes associated with perinatal brain injury such as apoptosis and inflammation, as well the possibility of it inducing increased turnover of the endogenous stem cell pools–neural, cardiac, renal, or muscle. However, many of the experiments discussed above were conducted *in vitro* and a few are conducted *in vivo. In vivo* testing is always the key, but given the multitude of cell and developmentally regulated effects of MK, it seems to have even greater importance to prove the validity of MK as a potential therapeutic. It will also be important to understand the roles of MK in animal models of development and injury, which incorporate the maternal–placental–fetal unit, such as the precocial spiny mouse (236, 237), or fetal and newborn sheep (165, 238). Current data predominantly comes from altricial species but given the high levels of MK in the amniotic fluid, there is the possibility that preterm birth causes an MK deficiency, and the effect of prematurity and brain injury in these infants warrants attention. Therefore, further *in vivo* investigations are needed to demonstrate the efficacy of MK, and improved drug delivery platforms are warranted to determine how its therapeutic efficacy and bioavailability can be fully realized in the context of perinatal brain injury.

## Author Contributions

All authors listed have made a substantial, direct and intellectual contribution to the work, and approved it for publication.

## Conflict of Interest

The authors declare that the research was conducted in the absence of any commercial or financial relationships that could be construed as a potential conflict of interest.
